# Supplementary material for: Hybrid Models and Biological Model Reduction with PyDSTool
Source: PLoS Comput Biol. 2012 Aug 9;8(8):e1002628. doi: 10.1371/journal.pcbi.1002628 (PMC3415397; doi:10.1371/journal.pcbi.1002628)
Supplement: Text S4 — Complete source code for the PyDSTool package (version 0.88.120504). Includes API documentation and help files linking to web pages. This file is identical to the current public release on Sourceforge.net. (ZIP) [file pcbi.1002628.s004.zip › PyDSTool/html/PyDSTool.Toolbox.dataanalysis-pysrc.html]

xml version="1.0" encoding="ascii"?


PyDSTool.Toolbox.dataanalysis


| Home | Trees | Indices | Help | PyDSTool |
| --- | --- | --- | --- | --- |

Package PyDSTool ::
Package Toolbox ::
Module dataanalysis

[hide private]

[frames] | no frames]

# Source Code for Module PyDSTool.Toolbox.dataanalysis

```
  1  """
 
  2  Data analysis utilities
 
  3  
 
  4  Robert Clewley, August 2006
 
  5  """ 
  6  
 
  7  from __future__ import division 
  8  from PyDSTool import * 
  9  from PyDSTool.common import _num_types, _int_types, _seq_types 
 10  from numpy import array, dot, zeros, transpose, shape, extract, mean, std 
 11  from numpy.linalg import norm 
 12  
 
 13  try: 
 14      from mdp.nodes import PCANode 
 15  except: 
 16      print "dataanalysis.py:: WARNING: MDP toolbox must be installed to use PCA" 
 17      print "You will get errors if you try to use any PCA-related functions" 
 18      print "  imported from this toolbox" 
 19      PCANode = None 
 20  from matplotlib.pylab import figure, plot, hold, polyfit, \
 
 21       bar, subplot, axes, title, xlabel, ylabel, draw, semilogy 
 22  from copy import copy 
 23  
 
 24  
 
 25  _PCA_utils = ['doPCA', 'get_evec', 'pca_dim', 'get_residual',
 
 26                'plot_PCA_residuals', 'plot_PCA_spectrum'] 
 27  
 
 28  _metric_utils = ['log_distances', 'log_distances_with_D'] 
 29  
 
 30  _analysis_utils = ['find_knees', 'whiten', 'find_closest_val', 'out_of_seq',
 
 31                     'find_nearby_ball', 'find_nearby_annulus', 'data_bins',
 
 32                     'find_central_point', 'find_diameter', 'find_from_sorted',
 
 33                     'dist_between_datasets', 'find_recurrences'] 
 34  
 
 35  _fit_utils = ['get_linear_regression_residual', 'fitline'] 
 36  
 
 37  __all__ = _PCA_utils + _metric_utils + _analysis_utils + _fit_utils 
 38  
 
 39  # -------------------------------------------------------------------------
 
 40  
 
 41  # Principal Component Analysis utilities
 
 42  
 


43 -def doPCA(in_pts, D, tol=.8):


44      pts=copy(in_pts) 
 45      pts.shape = (len(in_pts),D) 
 46      p=PCANode(output_dim=tol) 
 47      p.train(pts) 
 48      p.stop_training() 
 49      return p

 50  
 
 51  
 


52 -def get_evec(p, dim):


53      # extra call to array() to make it contiguous
 
 54      v = array(transpose(p.v)[dim-1]) 
 55      v.shape = (totdim, 3) 
 56      return v

 57  
 
 58  
 


59 -def pca_dim(pca, covering, data, refpt, tol=0.8):


60      if type(refpt) != int: 
 61          refpt = refpt[0] 
 62      pca[refpt] = doPCA(nhd(data, covering, refpt), tol) 
 63      print "PCA eigenvalues for nhd #%i:"%refpt 
 64      print pca[refpt].d 
 65      return len(pca[refpt].d)

 66  
 
 67  
 


68 -def get_residual(pcanode, pts):


69      pvals = pcanode.execute(pts, pcanode.get_output_dim()) 
 70      pproj = mat(pvals)*mat(transpose(pcanode.v)) 
 71      res = pts-array(pproj) # orthogonal complement of projection onto PCs 
 72      resval_part = norm(res) 
 73      resval = norm(resval_part) 
 74      return resval

 75  
 
 76  
 


77 -def plot_PCA_residuals(data, D=None, newfig=True, marker='o',
 
 78                         silent=False):


79      if D is None: 
 80          D = shape(data)[1] 
 81      p=doPCA(data, D, D) 
 82      spec=zeros((D,1),float) 
 83      for i in range(1,D): 
 84          spec[i] = norm(p.d[:i]) 
 85      res = transpose(sqrt(spec[-1]**2-spec**2)/spec[-1])[0] 
 86      if not silent: 
 87          print "L2-norm of PCA spectrum =", spec[-1] 
 88          if newfig: 
 89              figure() 
 90              style='k'+marker 
 91          else: 
 92              style=marker 
 93          semilogy(res,style) 
 94      ##    title('PCA residuals')
 
 95          xlabel(r'$\rm{Dimension}$',fontsize=20) 
 96          ylabel(r'$\rm{PCA \ residual}$',fontsize=20) 
 97      return p, res

 98  
 
 99  
 


100 -def plot_PCA_spectrum(data, D):


101      p=doPCA(data, D, D) 
102      spec=zeros((D,1),float) 
103      for i in range(1,D): 
104          spec[i] = norm(p.d[:i]) 
105      figure();plot(spec,'ko') 
106      title('L2-norm of spectrum of singular values') 
107      xlabel('D') 
108      ylabel('|s|') 
109      return p

110  
 
111  # -----------------------------------------------------------------------------
 
112  
 
113  
 


114 -def log_distances(m, sampleix=0, doplot=True, quiet=True, logv=None,
 
115                plotstyle=None, return_ixs=False):


116      """Log distances (L2-norm) of data points from a reference point (sampleix).
 
117      
 
118      For multiple calls to this function on the same data, it's faster to
 
119      pass logv precomputed.
 
120  
 
121      return_ixs=True also returns the point indices from the sort to
 
122      be returned in this argument in a third return value.
 
123      """ 
124      npts = size(m,0) 
125      assert sampleix >= 0 
126      d = zeros((npts-1,), 'd') 
127      if rank(m) == 3: 
128          for i in xrange(npts): 
129              if sampleix != i: 
130                  try: 
131                      d[i] = norm(m[sampleix,:,:]-m[i,:,:]) 
132                  except IndexError: 
133                      # catch case when index is too large for npts-1
 
134                      # so use the empty i=pix position (ordering doesn't matter)
 
135                      d[sampleix] = norm(m[sampleix,:,:]-m[i,:,:]) 
136      elif rank(m) == 2: 
137          for i in xrange(npts): 
138              if sampleix != i: 
139                  try: 
140                      d[i] = norm(m[sampleix,:]-m[i,:]) 
141                  except IndexError: 
142                      # catch case when index is too large for npts-1
 
143                      # so use the empty i=pix position (ordering doesn't matter)
 
144                      d[sampleix] = norm(m[sampleix,:]-m[i,:]) 
145      else: 
146          raise ValueError("Rank of input data must be 2 or 3") 
147      if return_ixs: 
148          # return sorted indices in that list argument
 
149          # (assumed to be an empty list)
 
150          ixs = array(argsort(d)) 
151          d = d[ixs] 
152      else: 
153          # just sort
 
154          d.sort() 
155      if not quiet: 
156          print "Chose reference point %i"%sampleix 
157          print "Min distance = %f, max distance = %f"%(d[0], d[-1]) 
158      logd = log(d).ravel() 
159      if logv is None: 
160          logv = log(range(1,len(d)+1)) 
161      nan_ixs = isfinite(logd)  # mask, not a list of indices 
162      logd = extract(nan_ixs, logd) 
163      logv = extract(nan_ixs, logv) 
164      if doplot: 
165          if plotstyle is None: 
166              plot(logd,logv) 
167          else: 
168              plot(logd,logv,plotstyle) 
169      if return_ixs: 
170          ixs = extract(nan_ixs, ixs) 
171          return (logv, logd, ixs) 
172      else: 
173          return (logv, logd)

174  
 
175  
 
176  # used for log_distances_with_D
 
177  dict_tol = array([1e-8]) 
178  
 
179  
 


180 -def log_distances_with_D(m, sampleix, logv=None, return_ixs=False):


181      """Log distances (L2-norm) of data points from a reference point (sampleix).
 
182      Additionally, it returns a list of the inter-point distances,
 
183      relative to the reference point, and the inverse of this mapping.
 
184  
 
185      For multiple calls to this function on the same data, it's faster to
 
186      pass logv precomputed.
 
187      
 
188      return_ixs=True returns the point indices from the sort in a fifth
 
189      return value. This identifies the points in the returned
 
190      list of inter-point distances and its inverse.
 
191      """ 
192      npts = size(m,0) 
193      dk = zeros((npts-1,), 'd') 
194      d_inv = {} 
195      # do in two stages: ix 0->sampleix, sampleix+1->npts-1
 
196      # but offset second stage indices in dk so there's a total of n-1
 
197      # this is basically a slightly unrolled loop to make it a little faster
 
198      if rank(m) == 3: 
199          for i in xrange(sampleix): 
200              dk[i] = norm(m[sampleix,:,:]-m[i,:,:]) 
201              if dk[i] in d_inv: 
202                  # ensure we find a unique distance for dictionary key
 
203                  # (doesn't matter if we adjust it at very fine resolution
 
204                  # to become unique)
 
205                  done = 0 
206                  u = dk[i] 
207                  sgn = 1 
208                  lim=40 
209                  while done < lim: 
210                      u = dk[i] + sgn*done*dict_tol 
211                      if u[0] not in d_inv: 
212                          done = lim+1  # avoids exception below 
213                      else: 
214                          if sgn == -1: 
215                              sgn = 1 
216                          else: 
217                              done += 1 
218                              sgn = -1 
219                  if done == lim: 
220                      raise ValueError("Non-unique distance found") 
221                  dk[i] = u 
222                  d_inv[u[0]] = i 
223              else: 
224                  d_inv[dk[i]] = i 
225          for i in xrange(sampleix+1, npts): 
226              ki = i-1 
227              dk[ki] = norm(m[sampleix,:,:]-m[i,:,:]) 
228              if dk[ki] in d_inv: 
229                  # ensure we find a unique distance for dictionary key
 
230                  # (doesn't matter if we adjust it at very fine resolution
 
231                  # to become unique)
 
232                  done = 0 
233                  u = dk[ki] 
234                  sgn = 1 
235                  lim=40 
236                  while done < lim: 
237                      u = dk[ki] + sgn*done*dict_tol 
238                      if u[0] not in d_inv: 
239                          done = lim+1  # avoids exception below 
240                      else: 
241                          if sgn == -1: 
242                              sgn = 1 
243                          else: 
244                              done += 1 
245                              sgn = -1 
246                  if done == lim: 
247                      raise ValueError("Non-unique distance found") 
248                  dk[ki] = u 
249                  d_inv[u[0]] = i 
250              else: 
251                  d_inv[dk[ki]] = i 
252      elif rank(m) == 2: 
253          for i in xrange(sampleix): 
254              dk[i] = norm(m[sampleix,:]-m[i,:]) 
255              if dk[i] in d_inv: 
256                  # ensure we find a unique distance for dictionary key
 
257                  # (doesn't matter if we adjust it at very fine resolution
 
258                  # to become unique)
 
259                  done = 0 
260                  u = dk[i] 
261                  sgn = 1 
262                  lim=40 
263                  while done < lim: 
264                      u = dk[i] + sgn*done*dict_tol 
265                      if u[0] not in d_inv: 
266                          done = lim+1  # avoids exception below 
267                      else: 
268                          if sgn == -1: 
269                              sgn = 1 
270                          else: 
271                              done += 1 
272                              sgn = -1 
273                  if done == lim: 
274                      raise ValueError("Non-unique distance found") 
275                  dk[i] = u 
276                  d_inv[u[0]] = i 
277              else: 
278                  d_inv[dk[i]] = i 
279          for i in xrange(sampleix+1, npts): 
280              ki = i-1 
281              dk[ki] = norm(m[sampleix,:]-m[i,:]) 
282              if dk[ki] in d_inv: 
283                  # ensure we find a unique distance for dictionary key
 
284                  # (doesn't matter if we adjust it at very fine resolution
 
285                  # to become unique)
 
286                  done = 0 
287                  u = dk[ki] 
288                  sgn = 1 
289                  lim=40 
290                  while done < lim: 
291                      u = dk[ki] + sgn*done*dict_tol 
292                      if u[0] not in d_inv: 
293                          done = lim+1  # avoids exception below 
294                      else: 
295                          if sgn == -1: 
296                              sgn = 1 
297                          else: 
298                              done += 1 
299                              sgn = -1 
300                  if done == lim: 
301                      raise ValueError("Non-unique distance found") 
302                  dk[ki] = u 
303                  d_inv[u[0]] = i 
304              else: 
305                  d_inv[dk[ki]] = i 
306  
 
307      else: 
308          raise ValueError("Rank of input data must be 2 or 3") 
309      d = copy(dk)  # dk remains unsorted 
310      if return_ixs: 
311          # return sorted indices in that list argument
 
312          # (assumed to be an empty list)
 
313          ixs = array(argsort(d)) 
314          d = d[ixs] 
315      else: 
316          # just sort
 
317          d.sort() 
318      logd = log(d).ravel() 
319      if logv is None: 
320          logv = log(range(1,len(d)+1)) 
321      nan_ixs = isfinite(logd)  # mask, not a list of indices 
322      logd = extract(nan_ixs, logd) 
323      logv = extract(nan_ixs, logv) 
324      d = extract(nan_ixs, d) 
325  ##    for i in range(len(d)):
 
326  ##        if nan_ixs[i] == 0:
 
327  ##            try:
 
328  ##                del d_inv[dk[i][0]]
 
329  ##                print "Deleted key %i @ %f"%(i,dk[i][0])
 
330  ##            except KeyError:
 
331  ##                print "Failed to delete key %i @ %f"%(i,dk[i][0])
 
332  ####                raise
 
333      if return_ixs: 
334          ixs = extract(nan_ixs, ixs) 
335          return (logv, logd, d, d_inv, ixs) 
336      else: 
337          return (logv, logd, d, d_inv)

338  
 
339  # ----------------------------------------------------------------------------
 
340  
 
341  # Data fitting utilities
 
342  
 


343 -def get_linear_regression_residual(pfit, x, y, weight='', w=0):


344      """Also see scipy.stat.linregress for a more sophisticated version
 
345      """ 
346      res = 0 
347      if weight == 'lo': 
348          for i in range(len(x)): 
349              res += ((y[i]-(pfit[0]*x[i]+pfit[1]))/(i+1)/w)**2 
350      elif weight == 'hi': 
351          l = len(x) 
352          for i in range(l): 
353              res += ((y[i]-(pfit[0]*x[i]+pfit[1]))/(l-i+1)/w)**2 
354      else: 
355          for i in range(len(x)): 
356              res += (y[i]-(pfit[0]*x[i]+pfit[1]))**2 
357      return sqrt(res)

358  
 
359  
 


360 -def fitline(x, y, lo=0, hi=1, doplot=True, quiet=True, linewidth=2):


361      """fitline takes the position of low and high fit limits and
 
362      returns the slope. Integer indices in the x data set can be
 
363      specified for the low and high limits, otherwise use a fraction
 
364      between 0 and 1.
 
365      
 
366      In application to fractal dimension estimation, if
 
367      x = log d (radius, a.k.a. inter-point distance) and
 
368      y = log v (index, a.k.a. estimate of volume at a given radius)
 
369      then the slope is the dimension estimate of the data set.
 
370  
 
371      Also see scipy.stat.linregress for a more sophisticated version
 
372      """ 
373      lendat = len(x) 
374      assert hi > lo 
375      if lo >= 0 and hi <= 1: 
376          loix = int(lendat*lo) 
377          hiix = int(lendat*hi) 
378      elif lo >=0 and hi > 0: 
379          loix = int(lo) 
380          hiix = int(hi) 
381      else: 
382          raise ValueError("Invalid low and high fit limits") 
383      if not quiet: 
384          print "lo ix %d, hi ix %d, max ix %d"%(loix, hiix, lendat-1) 
385      pfit = polyfit(x[loix:hiix],y[loix:hiix],1) 
386  #    print pfit
 
387      if doplot: 
388          plot([x[loix],x[hiix]],
 
389               [x[loix]*pfit[0]+pfit[1],x[hiix]*pfit[0]+pfit[1]],
 
390               linewidth=linewidth) 
391      return pfit[0]

392  
 
393  
 
394  
 
395  # ----------------------------------------------------------------------------
 
396  
 
397  # Data analysis utilities
 
398  
 


399 -class data_bins(object):


400      """Class for data binning. Indexed by bin number 0 - (num_bins-1).
 
401  
 
402      Initialization arguments:
 
403              coordinate -- name of coordinate that occupies bins
 
404              bin_ords   -- ordered sequence of n bin ordinates (integers or floats)
 
405                            defining n-1 bins
 
406              valuedict  -- optional dictionary containing initial values for
 
407                            bins: keys = ordinate values,
 
408                                  values = coordinate values
 
409              tolerance  -- for resolving bin edges with finite precision
 
410                            arithmetic (defaults to 1e-12)
 
411      """ 
412      
 


413 -    def __init__(self, coordinate, bin_ords, valuedict=None, tolerance=1e-12):


414          assert isincreasing(bin_ords), \
 
415                  "Bin ordinates must be given as a sequence in increasing order" 
416          # bins are referenced internally by integer index
 
417          self.num_bins = len(bin_ords)-1 
418          assert self.num_bins > 0, "Must be more than 2 ordinates to define bins" 
419          self.bin_ords = array(bin_ords) 
420          self.bins = dict(zip(range(self.num_bins),[0]*self.num_bins)) 
421          self.coordinate = coordinate 
422          self.tolerance = tolerance 
423          self.intervals = [Interval('bin_%s'%i, float,
 
424                                  (bin_ords[i],bin_ords[i+1]), \
 
425                                  abseps=tolerance) for i in range(self.num_bins)] 
426          self.midpoints = (self.bin_ords[1:]+self.bin_ords[:-1])/2. 
427          if valuedict is not None: 
428              for k, v in valuedict.iteritems(): 
429                  self[self.resolve_bin_index(k)] = v

430  
 


431 -    def resolve_bin_index(self, ordinate):


432          """Find bin number (index) associated with ordinate""" 
433          try: 
434              # return first bin index for which ordinate in bin interval is
 
435              # 'uncertain' or 'contained'
 
436              return [interval.contains(ordinate) is not notcontained \
 
437                         for interval in self.intervals].index(True) 
438          except ValueError: 
439              # no bin found
 
440              raise ValueError("No corresponding bin for this ordinate")

441  
 


442 -    def __call__(self, ordinate):


443          """Return content of the bin that ordinate resolves to.
 
444          """ 
445          return self.bins[self.resolve_bin_index(ordinate)]

446  
 


447 -    def __getitem__(self, bin_index):


448          if isinstance(bin_index, _int_types): 
449              # bin_index is a single index, so...
 
450              # return first bin for which ordinate in bin interval is
 
451              # 'uncertain' or 'contained'
 
452              return self.bins[bin_index] 
453          elif isinstance(bin_index, slice): 
454              if bin_index.step is not None: 
455                  if bin_index.step != 1: 
456                      raise ValueError("Cannot step in slice of bins") 
457              if bin_index.start is None: 
458                  start = 0 
459              else: 
460                  start = bin_index.start 
461              if bin_index.stop is None: 
462                  stop = self.num_bins 
463              else: 
464                  stop = bin_index.stop 
465              assert start < stop, \
 
466                         "Slice must go in increasing order" 
467              new_bin_ords = self.bin_ords[start:stop+1] 
468              b = bin(self.coordinate, new_bin_ords,
 
469                      tolerance=self.tolerance) 
470              for x in new_bin_ords[:-1]: 
471                  bin_ix = self.resolve_bin_index(x+self.tolerance) 
472                  b.bins[bin_ix] = self.bins[bin_ix] 
473              return b 
474          else: 
475              raise ValueError("Invalid indexing of bins")

476  
 


477 -    def __setitem__(self, bin_index, value):


478          """Set value of a given bin number (index).""" 
479          self.bins[bin_index] = value

480  
 


481 -    def increment(self, ordinate, inc=1):


482          """Increment the bin corresponding to the ordinate argument.
 
483          Optional argument inc sets increment amount (default 1).
 
484          """ 
485          self.bins[self.resolve_bin_index(ordinate)] += inc

486  
 


487 -    def bin(self, values):


488          """Increment bin contents according to values passed.""" 
489          if isinstance(values, _seq_types): 
490              for v in values: 
491                  self.increment(v) 
492          else: 
493              self.increment(values)

494  
 


495 -    def clear(self):


496          """Reset all bins to have zero contents.""" 
497          self.bins = dict(zip(range(self.num_bins),[0]*self.num_bins))

498  
 


499 -    def __str__(self):


500          return "Binned data with intervals %s:\n By bin number: %s"%(str([i.get() \
 
501                       for i in self.intervals]), str(self.bins))

502  
 
503      __repr__ = __str__ 
504  
 


505 -    def to_pointset(self):


506          """Convert to a pointset""" 
507          bin_ixs = xrange(self.num_bins) 
508          return Pointset(indepvararray=range(self.num_bins),
 
509                          coorddict={self.coordinate: [self.bins[ix] \
 
510                                                          for ix in bin_ixs],
 
511                                     'bin_limit_lo': [self.intervals[ix].get(0) \
 
512                                                          for ix in bin_ixs],
 
513                                     'bin_limit_hi': [self.intervals[ix].get(1) \
 
514                                                          for ix in bin_ixs]
 
515                                      })

516  
 


517 -    def to_arrays(self):


518          """Convert to two arrays of bin indices and values""" 
519          x, y = sortedDictLists(self.bins, byvalue=False) 
520          return (array(x), array(y))

521  
 


522 -    def values(self):


523          return sortedDictValues(self.bins)

524  
 


525 -    def keys(self):


526          return sortedDictKeys(self.bins)

527          
 


528 -    def mean(self):


529          """Mean of binned data""" 
530          a = self.midpoints*array(sortedDictValues(self.bins)) 
531          return sum(a) / sum(self.bins.values())

532  
 


533 -    def std(self):


534          """Standard deviation of binned data""" 
535          vals = array(sortedDictValues(self.bins)) 
536          a = self.midpoints*vals 
537          sum_bin_vals = sum(self.bins.values()) 
538          mean_val = sum(a) / sum_bin_vals 
539          return sqrt(sum(((self.midpoints-mean_val)*vals)**2) / sum_bin_vals)

540  
 
541  
 


542 -def find_closest_val(x, a, eps=1e-7, which_norm=2):


543      """Find one value in a closer than eps to x""" 
544      for aval in a: 
545          if norm(x-aval, which_norm) < eps: 
546              return aval 
547      return None

548  
 
549  
 


550 -def find_nearby_ball(data, refix, r, which_norm=2, include_ref=False):


551      """Return indices of all points in data that are inside a ball of
 
552      radius r from the reference point (not included).""" 
553      nearby = [] 
554      refpt = data[refix] 
555      if include_ref: 
556          for i in xrange(size(data,0)): 
557              if norm(refpt-data[i], which_norm) <= r: 
558                  nearby.append(i) 
559      else: 
560          for i in xrange(size(data,0)): 
561              if i != refix: 
562                  if norm(refpt-data[i], which_norm) <= r: 
563                      nearby.append(i) 
564      return nearby

565  
 


566 -def find_nearby_annulus(data, refix, rlo, rhi, which_norm=2):


567      """Return a list containing input data's indices of all neighbours of the
 
568      reference point within the range of distances d such that dlo < d < dhi
 
569      """ 
570      assert rlo > 0 and rhi > rlo 
571      nearby = [] 
572      for i in xrange(size(data,0)): 
573          if i != refix: 
574              d = norm(data[refix]-data[i], which_norm) 
575              if d > rlo and d < rhi: 
576                  nearby.append(i) 
577      return nearby

578  
 


579 -def find_central_point(data, which_norm=2):


580      """Find the index of a point closest to the 'centre' of a data set.
 
581      The centre is defined to be where the mean of the points' position
 
582      vectors relative to the centre is minimized in each direction.""" 
583      dim = data.shape[1] 
584      centre = mean(data,0) 
585      ds = array([norm(p, which_norm) for p in data-centre]) 
586      ds_sort_ixs = ds.argsort() 
587      return ds_sort_ixs[0]

588  
 
589  
 


590 -def find_recurrences(data, refix, r, times=None, ignore_small=0,
 
591                       which_norm=2):


592      """Find recurrences of a trajectory in a ball of radius r centred at the
 
593      reference point given by refix in data.
 
594  
 
595      If recurrence times are desired, pass an associated time array corresponding
 
596      to the points in the data.
 
597  
 
598      If ignore_small > 0, recurrences of fewer than this number of data points
 
599         will be treated as anomalous and ignored completely.
 
600  
 
601      Returns a structure (class common.args) having attributes:
 
602          partitions -- pairs of entry and exit indices for each recurrence in
 
603                  the ball
 
604          ball_ixs -- all consecutive indices into data for each recurrence
 
605          partitions_lengths -- number of indices in each partition
 
606          rec_times -- times to next recurrence in the set from previous
 
607      """ 
608      if times is not None: 
609          assert len(times) == len(data), "Times must have same length as data" 
610      # ball_ixs is already sorted
 
611      ball_ixs = find_nearby_ball(data, refix, r, which_norm, include_ref=True) 
612      result = args(ball_ixs=[], partitions=[], partition_lengths=[],
 
613                    rec_times=None) 
614      if len(ball_ixs) == 0: 
615          # No points found in ball
 
616          return result 
617      # find boundaries of each partition between contiguous indices
 
618      partition_bds = out_of_seq(ball_ixs) 
619      if len(partition_bds) == 0: 
620          result.ball_ixs = ball_ixs 
621          result.partitions = [(ball_ixs[0], ball_ixs[-1])] 
622          result.partition_lengths = [ball_ixs[-1] - ball_ixs[0] + 1] 
623          if times is not None: 
624              # only update this if times present to be consistent with other
 
625              # changes of rec_times from default None
 
626              result.rec_times = [Inf] 
627          return result 
628      else: 
629          plen = ball_ixs[partition_bds[0]-1] - ball_ixs[0] + 1 
630          if plen > ignore_small: 
631              partitions = [(ball_ixs[0], ball_ixs[partition_bds[0]-1])] 
632          else: 
633              partitions = [] 
634      # find contiguous indices (and corresponding times)
 
635      for i in range(len(partition_bds)-1): 
636          plo = ball_ixs[partition_bds[i]] 
637          phi = ball_ixs[partition_bds[i+1]-1] 
638          plen = phi - plo + 1 
639          if plen > ignore_small: 
640              partitions.append((plo, phi)) 
641      plo = ball_ixs[partition_bds[-1]] 
642      phi = ball_ixs[-1] 
643      plen = phi - plo + 1 
644      if plen > ignore_small: 
645          partitions.append((plo, phi)) 
646      result.ball_ixs = ball_ixs 
647      result.partitions = partitions 
648      result.partition_lengths = [p[1]-p[0]+1 for p in partitions] 
649      if times is None: 
650          return result 
651      else: 
652          result.rec_times = recurrence_times(times, partitions) 
653          return result

654  
 
655  
 


656 -def find_diameter(data, eps, which_norm=2):


657      """Find approximate diameter of data set, up to tolerance defined by eps > 0.
 
658  
 
659      Data assumed to be a rank-2 array.
 
660      """ 
661      assert eps > 0, "Tolerance must be positive" 
662      num_pts = len(data) 
663      if num_pts > 400: 
664          # If data well distributed, assume that 100 sample points is a good
 
665          # initial set to test
 
666          sample_rate = int(ceil(num_pts/100.)) 
667      else: 
668          # small enough data set to do by brute force on all points
 
669          sample_rate = 1 
670      old_diam = -Inf 
671      delta = Inf  # change in estimate of radius on each iteration 
672      while delta > eps: 
673          #print "Sample rate = ", sample_rate
 
674          downsampled = data[::sample_rate] 
675          mind, diam = dist_between_datasets(downsampled, downsampled,
 
676                                               which_norm) 
677          if sample_rate == 1: 
678              # can go no further!
 
679              delta = 0 
680              break 
681          else: 
682              sample_rate = int(ceil(sample_rate/2.)) 
683          delta = diam - old_diam 
684          old_diam = diam 
685          #print old_diam, delta
 
686      return diam

687  
 
688  
 


689 -def dist_between_datasets(data1, data2, which_norm=2):


690      maxd = -Inf 
691      mind = Inf 
692      for v1 in data1: 
693          for v2 in data2: 
694              d = norm(v1-v2, which_norm) 
695              if d > maxd: 
696                  maxd = d 
697              elif d < mind: 
698                  mind = d 
699      return (mind, maxd)

700  
 
701  
 


702 -def out_of_seq(a, inc_only=False):


703      """Determine if and where an integer array a is not increasing or
 
704      decreasing in sequence by 1.
 
705      """ 
706      v_old = a[0] 
707      i = 0 
708      out = [] 
709      dirn = 0 
710      for v in a[1:]: 
711          i +=1 
712          if v == v_old+1: 
713              if dirn == -1: 
714                  out.append(i) 
715              dirn = 1 
716          elif v == v_old-1: 
717              if dirn == 1: 
718                  out.append(i) 
719              dirn = -1 
720          else: 
721              dirn = 0 
722              out.append(i) 
723          v_old = v 
724      return out

725  
 
726  
 


727 -def whiten(data):


728      """Subtract mean and divide by standard deviation in each column of data""" 
729      wdata=zeros(shape(data),Float) 
730      for d in range(shape(data)[1]): 
731          x = data[:,d] 
732          wdata[:,d] = (x-mean(x))/std(x) 
733      return wdata

734  
 
735  
 


736 -def find_knees(data, tol=1., inlog=False, verbose=False):


737      """
 
738      Find one or more 'knees' in data, according to high second derivatives.
 
739      
 
740      inlog option finds knees in the logs of the data entries.
 
741      tol=1. works well if inlog=False
 
742      tol=0.3 works well if inlog=True
 
743      """ 
744      knee_ixs = [] 
745      for i in range(1,len(data)-1): 
746          if inlog: 
747              frac = log(data[i+1])+log(data[i-1])-2*log(data[i]) 
748          else: 
749              d2 = data[i+1]+data[i-1]-2*data[i] 
750              try: 
751                  frac = d2/data[i] 
752              except ZeroDivisionError: 
753                  frac = Inf 
754          if verbose: 
755              print i, data[i], frac 
756          if frac > tol and frac < Inf: 
757              knee_ixs.append((i,frac)) 
758      if verbose: 
759          print "High second derivatives at: ", knee_ixs, "\n" 
760      knees = [] 
761      found = False  # in a contiguous segment of high second derivatives 
762      curr_kixs = [] 
763      old_kix = None 
764      for i in range(len(knee_ixs)): 
765          process = False 
766          kix, frac = knee_ixs[i] 
767          if verbose: 
768              print "Processing knee at index", kix 
769          if kix-1 == old_kix: 
770              if i == len(knee_ixs)-1: 
771                  # this is the last index so have to process
 
772                  process = True 
773              if found: 
774                  curr_kixs.append(i) 
775              else: 
776                  curr_kixs = [i-1, i] 
777                  found = True 
778          else: 
779              process = old_kix is not None 
780          if verbose: 
781              print old_kix, found, curr_kixs 
782          if process: 
783              if found: 
784                  found = False 
785                  if verbose: 
786                      print "Current knee indices:", curr_kixs, 
787                      print [knee_ixs[k] for k in curr_kixs] 
788                  all_d2 = array([knee_ixs[k][1] for k in curr_kixs]) 
789                  ixs_sort = argsort(all_d2) 
790                  max_ix = ixs_sort[-1] 
791                  knees.append(knee_ixs[curr_kixs[max_ix]][0]) 
792                  curr_kixs = [] 
793              else: 
794                  if verbose: 
795                      print "Appending knee index", old_kix 
796                  knees.append(old_kix) 
797          old_kix = kix 
798      # add final singleton index of high derivative, if any
 
799      if not found and old_kix not in knees: 
800          if verbose: 
801              print "Appending knee index", old_kix 
802          knees.append(old_kix) 
803      return knees

804  
 
805  
 
806  # -----------------------------------------------------------------------------
 
807  
 
808  # Internal helper functions
 
809  
 


810 -def recurrence_times(ts, partitions):


811      """Internal function for use by find_recurrences""" 
812      rtimes = [] 
813      if len(partitions) == 1: 
814          return [Inf] 
815      for i in range(len(partitions)-1): 
816          (plo, phi) = partitions[i] 
817          rtimes.append(ts[partitions[i+1][0]]-ts[partitions[i][1]]) 
818      return rtimes

819  
 
820  
 


821 -def find_from_sorted(x, v, next_largest=1, indices=None):


822      if isinstance(v, _seq_types): 
823          # sequence v
 
824          return [find(array(x), y, next_largest, indices) for y in v] 
825      else: 
826          # singleton v
 
827          return find(array(x), v, next_largest, indices)

828  
 


829 -def colormap(mag, cmin, cmax):


830      """
 
831      Return a tuple of floats between 0 and 1 for the red, green and
 
832      blue amplitudes.
 
833      Function originally named floatRgb and written by Alexander Pletzer,
 
834      from the Python Cookbook.
 
835      """ 
836  
 
837      try: 
838            # normalize to [0,1]
 
839            x = float(mag-cmin)/float(cmax-cmin) 
840      except: 
841            # cmax = cmin
 
842            x = 0.5 
843      blue = min((max((4*(0.75-x), 0.)), 1.)) 
844      red  = min((max((4*(x-0.25), 0.)), 1.)) 
845      green= min((max((4*math.fabs(x-0.5)-1., 0.)), 1.)) 
846      return (red, green, blue)

847  
 
848  
 


849 -def dump_progress(x, tot, filename='progress_info.dat'):


850      """Send progress report to a file""" 
851      f=open(filename, 'w') 
852      f.write("Progress: %d / %d \n"%(x,tot)) 
853      f.write("Date and time of update: " + " ".join([str(x) for x in localtime()[:-3]]) + "\n\n") 
854      f.close()

855  
 
856  
 


857 -def second_diff(data, i):


858      """Naive second difference formula at position i of data""" 
859      return data[i+1]+data[i-1]-2*data[i]

860  
 
861  
 


862 -def _adjust_ix(i, n):


863      """Internal helper function""" 
864      if i >= n: 
865          return i+1 
866      else: 
867          return i

868
```

  


| Home | Trees | Indices | Help | PyDSTool |
| --- | --- | --- | --- | --- |

|  |  |
| --- | --- |
| Generated by Epydoc 3.0.1 on Fri May 4 15:24:12 2012 | http://epydoc.sourceforge.net |
